# Development and Evaluation of Integrated Chrono-Nutrition Weight Reduction Program among Overweight/Obese with Morning and Evening Chronotypes

**DOI:** 10.3390/ijerph19084469

**Published:** 2022-04-07

**Authors:** Fatin Hanani Mazri, Zahara Abdul Manaf, Suzana Shahar, Arimi Fitri Mat Ludin, Siti Munirah Abdul Basir

**Affiliations:** 1 Dietetic Program and Centre for Healthy Aging and Wellness, Faculty of Health Sciences, Universiti Kebangsaan Malaysia, Jalan Raja Muda Abdul Aziz, Kuala Lumpur 50300, Malaysia; fatinhananimazri@gmail.com (F.H.M.); suzana.shahar@ukm.edu.my (S.S.); sitimunirah.abdulbasir@gmail.com (S.M.A.B.); 2 Biomedical Science Program and Centre for Healthy Aging and Wellness, Faculty of Health Sciences, Universiti Kebangsaan Malaysia, Jalan Raja Muda Abdul Aziz, Kuala Lumpur 50300, Malaysia; arimifitri@ukm.edu.my

**Keywords:** chrono-nutrition, weight reduction, chronotypes, temporal eating pattern, meal timing, sleep

## Abstract

This paper describes the development of an integrated chrono-nutrition weight reduction program and the evaluation of the attendance, retention, satisfaction and compliance towards the chrono-nutrition components among morning and evening chronotypes for overweight/obese non-shift workers. The present study was conducted in two phases: Phase I was composed of needs assessments on the chronotypes’ dietary patterns and chrono-nutrition through a scoping review and integrating the chrono-nutrition components (temporal eating pattern, meal timing and sleeping habits) alongside the existing weight reduction module, SLIMSHAPE™. Phase II consisted of a feasibility study to evaluate the integrated chrono-nutrition weight reduction program (SLIMSHAPE™ Chrono). A total of 91 overweight/obese non-shift workers participated in the 12-week weight reduction program (Age: 39.6 ± 6.3 years; 74.7% women; BMI: 31.2 ± 4.5 kg/m^2^). Low attrition rate was recorded, with 85 participants (93.4%) completing the pre- and post-intervention assessments. Overall, morning and evening chronotypes had increased their % energy intake in the early eating window (MT: 64.8 vs. 67.2%, ET: 62.7 vs. 65.6%, Mean difference (MD): 2.8, 95%CI: 0.3, 5.1, *p* = 0.028) and reduced their intake in the late eating window (MT: 35.2 vs. 32.8%, ET: 37.3 vs. 34.4%, MD: −2.8, 95%CI: −5.1, −0.3, *p* = 0.028) and earlier midpoint of eating (MT: 14:02 vs. 13:49; ET: 14:27 vs. 14:18, 95%CI: −0.4, −0.02, *p* = 0.029) and had a reduced night eating syndrome score (MT: 10.0 vs. 8.9; ET: 10.7 vs. 8.9, MD: −1.5, 95%CI: −2.5, −0.5, *p* = 0.004). There was no significant change in the first (MT: 08:12 vs. 08:04, ET: 08:24 vs. 08:22, MD: −0.1, 95%CI: −0.2, 0.03, *p* = 0.170) and last mealtime (MT: 19:52 vs. 19:33, ET: 20:29 vs. 20:14, MD: −0.3, 95%CI: −0.6, −0.04, *p* = 0.081), eating duration (MT: 11.7 vs. 11.5 h, ET: 12.1 vs. 11.9 h, MD: −0.2, 95%CI: −0.6, 0.2, *p* = 0.251) and the elapse time between sleep onset and last meal (MT: 3.1 vs. 3.5 h, ET: 3.5 vs. 3.2 h, MD: 0.1, 95%CI: −0.3, 0.4, *p* = 0.678). In terms of sleep, evening chronotypes increased their sleep duration (MD: 0.8 h, 95% CI: 0.4, 1.2, *p* < 0.001) and reduced social jetlag (MD: 19 min, 95% CI: 1.7, 36.3, *p* = 0.031) post-intervention compared to morning chronotypes. The integrated chrono-nutrition weight reduction program among morning and evening chronotypes improved the temporal pattern of energy intake, meal timing, night eating syndrome and sleep habits post-intervention. The chrono-nutrition practice could be a potentially modifiable behavior as an adjunct strategy in weight management.

## 1. Introduction

Obesity is a global health threat linked to numerous comorbidities, including diabetes mellitus, cardiovascular disease, obstructive sleep apnea, gastrointestinal, joint and muscular disorders, cancers and psychological problems [1]. In 2017, 4.7 million deaths of noncommunicable disease were associated with high body mass index (BMI), and it was estimated that the number would rise to 5.5 million in 2025 [2]. Moreover, obesity impairs the immune response and increases the risks of severe COVID-19 symptoms [3]. Obesity also affects the economy at individual and nation levels, with increases in healthcare expenditure and losses of productivity at work [4]. Therefore, various strategies have been implemented to combat obesity, including nutrition, physical activity, behavioral lifestyle, pharmacology, and surgical interventions [5,6,7,8]. In terms of dietary intervention, calorie restriction is primarily the main component to induce weight loss [9]. In addition, emerging research to demonstrate the plausible role of chrono-nutrition in obesity is ongoing [10].

Chrono-nutrition encompasses meal timing and temporal pattern study of daily intake and its relation to the circadian rhythm [11]. Late mealtime is linked to greater adiposity [12,13] and reduced weight intervention efficacy [14]. Experimental studies on temporal eating patterns demonstrate that greater energy intake during earlier meals (breakfast and lunch) results in greater weight loss as compared to greater energy intake during dinner [15,16,17]. However, these studies did not explore the association to the chronotype. A study by Xiao and colleagues (2019) [18] discovered that greater consumption after waking up and reduced consumption close to bedtime was associated with lower risk of obesity only among the morning chronotype, which implied the possible interaction of chronotype in temporal eating patterns. In addition, the evening chronotype was associated with later mealtime [19,20] and greater consumption at night compared to the morning chronotype [21]. Furthermore, evening chronotypes were associated with obesity in observational studies [19,22,23,24,25], but there was limited evidence in experimental studies. Muñoz et al. (2019) [26] conducted a randomized trial on chronotype-adjusted diet for weight reduction, in which morning and evening chronotypes received a specific prescription of chronotype temporal energy intake distribution, with more energy deposited in the earlier part of the day. Although the author reported a significant weight loss among the participants who received the chronotype-tailored diet than the control participants, specific information on the actual dietary practice, including dietary intake, temporal energy intake, meal timing and sleep, are not discussed in great detail. On the other hand, a recent short weight intervention—3 weeks in a hospital-controlled setting—prescribed early meal and sleep timing patterns in addition to moderate calorie restriction for both morning and evening chronotypes [27]. Evening chronotypes showed a greater weight loss trend as compared to morning chronotypes, possibly due to evening chronotypes exerting greater change in their meal timing and indirectly shifting greater calorie intake towards earlier of the day. However, there is insufficient information on the changes in mealtime and the temporal energy intake related to their baseline practice. Nonetheless, the replication of this study in a free-living environment is required to assess the adherence towards early meal and sleep timing, particularly among evening chronotypes.

Although extensive research has been carried out on meal timing and temporal energy patterns, there have been limited studies incorporating both aspects of chrono-nutrition in a single weight reduction intervention to assess the compliance and response towards the dietary prescriptions. Moreover, sleep deficiency and irregularity was associated with circadian disruption [28,29], greater BMI [30] and increased daily calorie intake [31]; thus, these factors have the potential to be included as a part of a holistic approach to chrono-nutrition weight intervention. In addition, there have been limited local reported data on temporal energy intake patterns, such as energy intake according to meal occasions among working adults. Previous studies have focused more on dietary patterns of students (school or university) [32] or on meal frequency [33]. Hence, this study aims to develop an integrated chrono-nutrition weight reduction program and evaluate the attendance, retention, satisfaction and compliance towards the chrono-nutrition components among morning and evening chronotypes in overweight/obese non-shift workers. Furthermore, we also describe the adiposity and biochemical parameter outcomes upon completing of this program.

## 2. Materials and Methods

This is a quasi-experimental study design consisting of two phases: (1) development of integrated chrono-nutrition weight reduction program and (2) evaluation of the integrated program through a feasibility study. This study was conducted in accordance with the Declaration of Helsinki and approved by the Research and Ethical Committee of Medical Research of Universiti Kebangsaan Malaysia (UKM PPI/111/8/JEP-2017-656).

*Phase 1: Development of Integrated Chrono-Nutrition Weight Reduction Program*

### 2.1. Needs Assessments of Chrono-Nutrition Domains

An extensive search was conducted on observational and intervention evidence related to chronotype dietary patterns among adults, including dietary behavior, nutrients, and food intake. Based on the scoping review, two domains of chrono-nutrition are distinctly different between morning and evening chronotypes (i) temporal pattern of energy intake distribution and (ii) meal timing [34]. A further literature search on intervention studies related to the temporal pattern of energy intake and meal timing was conducted, including studies that did not assess chronotype to elucidate the intervention approach. Previous intervention studies that implemented temporal energy patterns suggested alteration of energy intake during main meals (breakfast, lunch or dinner) and prescribed greater energy intake during the earlier meals of the day and less intake during later meals of the day [15,16,26]. Distribution of energy intake implemented in a study by Muñoz et al. (2019) was adopted in our module (Table 1). The energy prescription was moderately distributed throughout all meal occasions and specifically designed for morning and evening chronotypes. As for mealtime and sleep, the recommendations proposed by Lopez-Minguez et al. (2016) [35] and the National Sleep Foundation [36] were adopted and adapted.

### 2.2. Integration of Chrono-Nutrition and SLIMSHAPE™ Weight Reduction Module

The proposed chrono-nutrition domains were reviewed and incorporated into the existing workplace weight reduction module, SLIMSHAPE™ [37,38] which was developed based on the Health Belief Model [39]. The SLIMSHAPE™ program was delivered face to face through talks, demonstrations, interactive activities and group activities conducted by a group of multidisciplinary practitioners including dietitians, an exercise physiologist, a psychologist, and a physician. This program was held on work days (14:30 to 17:00) and was in the vicinity of participants’ workplace.

The newest integrated chrono-nutrition weight reduction program is named SLIMSHAPE™ Chrono. Table 2 shows the similarities and differences in intervention components between the original SLIMSHAPE™ module and SLIMSHAPE™ Chrono module. The integrated module consisted of a 12-week (2 ½ h per session, once a week), face-to-face workplace weight reduction program. The SLIMSHAPE™ Chrono module adds the temporal prescription of energy intake based on early and late eating windows tailored to the chronotype of the participants. The temporal eating window was divided into the early eating window (early window) and the late eating window (late window), which was determined based on the midpoint of eating. The midpoint of eating is the midpoint between the first and last mealtime in a day [14]. For example, if the first mealtime is 08:00 and the last mealtime is 20:00, the midpoint of the eating window is 14:00. The early window refers to the timing before the midpoint of eating, which includes breakfast, morning snack and lunch. On the other hand, the late window refers to the timing after the midpoint of eating, which includes afternoon snack and dinner. At the beginning of SLIMSHAPE™ Chrono, participants received a meal plan, mealtime and sleep prescription. The meal plan, ranging from 1200 to 1800 kcal, were prepared based on the temporal energy distribution tailored for morning or evening chronotypes (Table 2). The meal plan consisted of the amount in each food group required for each meal (breakfast, lunch, dinner and snack), examples of food items in each food group and the portion size. In addition, dietitians guided the participants in meal planning and educated on food portion size. The other dietary intervention components, including healthy eating guides and cooking demonstrations as well as physical activity and behavior components, were delivered during the weekly session throughout the 12-week weight intervention program.

*Phase 2 Feasibility of Integrated Chrono-Nutrition Weight Reduction Program*

A feasibility study was conducted to test the integrated chrono-nutrition weight reduction module. The study was conducted among adults that lived and worked in Putrajaya, Malaysia. Putrajaya is an urban area in the center of Malaysia, which contains most of the major government offices. Putrajaya was chosen as the study location because this study targeted participants who are non-shift workers, which are common among government officers. The study was held between October 2019 and December 2019 (12 weeks). A written informed consent was obtained from each participant prior to the study. The inclusion criteria for this program were non-shift workers (1) aged 20–59 years; (2) body mass index (BMI) ≥ 25 kg/m^2^; (3) do not have chronic diseases such as cancer or renal and heart diseases; and (4) is at least at the contemplation stage of change. The participants were excluded if (1) pregnant or breastfeeding; (2) participated in any weight loss program within 3 months prior to this study or taking any medication/product for weight reduction; (3) has serious joint pain; (4) has uncontrolled diabetes mellitus (HbA1c > 9.0%) and hypertension (systolic blood pressure ≥ 160 mmHg or diastolic blood pressure ≥ 100 mmHg); and (5) shift worker.

**Figure 1:**
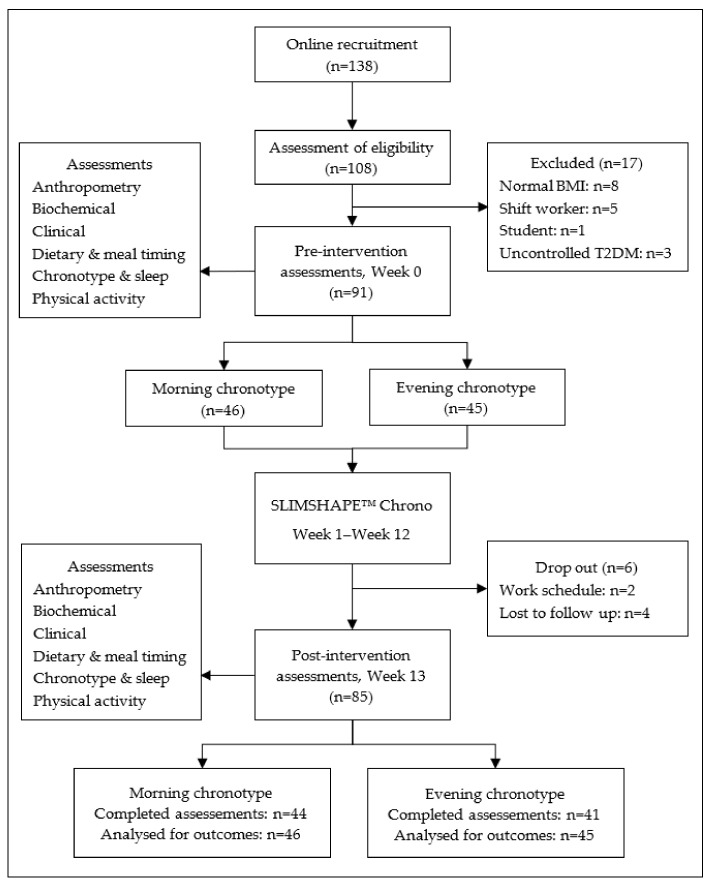
Flowchart of the SLIMSHAPE™ Chrono.

The sample size for the feasibility study was determined using GPower version 3.1. The calculation sample size was based on an F-test, with the f effect size calculated based on mean and standard deviation weight change from Muñoz et al. (2019) [26], with an alpha level of 0.05 and a test power of 80%, resulting in 88 participants. After considering 20% for possible losses (based on a 3-month drop-out rate in Muñoz et al. (2019)), a total of 105 participants were required. Figure 1 describes the flowchart of the SLIMSHAPE™ Chrono. The recruitment process started in August 2019–September 2019. The information about the weight reduction program was spread via online, posters, email and social media among the Putrajaya community. A total of 138 potential participants registered; however, only 108 turned up for the screening assessments. The final analysis included 91 participants. Seventeen participants were excluded because of normal BMI (*n* = 8), being shift workers (*n* = 5), on postgraduate study leave (*n* = 1) and uncontrolled diabetes mellitus type 2 (*n* = 3).

### 2.3. Measurement

The primary outcome of this feasibility study was attendance, satisfaction and retention rate and improvement in chrono-nutrition domains. The secondary outcome was changes in adiposity and biochemical parameters. All the assessments were measured during pre- and post-intervention (Figure 1).

#### 2.3.1. Socio-Demographic Background

The information on socio-demographic background, including age, gender, race, education level, marital status, monthly household income and self-reported medical history, was gathered. Based on the 2019 Household Income and Basic Survey Amenities report by the Department of Statistic Malaysia, the monthly household income (in Ringgit Malaysia, RM) was categorized into three income groups: low (<RM 4850), middle (RM 4850–10,970) and high (>RM 10,970) [40].

#### 2.3.2. Attendance and Satisfaction

The attendance for each session was recorded. Participants were considered compliant if they attended 9 out of the 12 sessions. The satisfaction evaluation was assessed using close-ended questions on the relevance of each session and whole program and open-ended questions on suggestions for improvement or the participants’ feedback on the program. The closed-ended responses were assessed using a Likert-scale score ranging from 1 (very poor) to 5 (very good).

#### 2.3.3. Chronotypes and Sleep Parameters

The chronotypes and sleep parameters such as sleep–wake timing, sleep duration and social jetlag were assessed using the Munich Chronotype Questionnaire (MCTQ) [41], modified for split-sleep and non-shift workers, which was validated prior to the current study [42]. Participants were dichotomized by sample population median midpoint of sleep on free days (corrected sleep debt) into morning (before 03:03) and evening (after 03:03) chronotypes [18]. The average sleep duration = ((sleep duration on work days × 5) + (sleep duration on free days × 2)) ÷ 7. The social jetlag = midpoint of sleep on free days − midpoint of sleep on work days [41].

#### 2.3.4. Dietary Information

The compliance towards the temporal eating pattern and meal timing was assessed using the 7-day diet history questionnaire (DHQ), validated for Malaysian population [43]. Participants were asked to recall their regular dietary intake. The household measurements were used to assist the participants to visualize their portion size estimation, and they were interviewed by trained dietitians/nutritionists. The nutrient intake was then analyzed using the Nutritionist Pro™ software, and the Malaysia Food Composition database was chosen when analyzing the nutrient intake (Axxya Systems, Woodinville, WA, USA). Participants were also asked the average timing of each meal reported. The eating window refers to the total eating duration from the first to last mealtime. The temporal patterns of energy and macronutrient intake were classified into early window (breakfast, morning snack and lunch) and late window (afternoon snack and dinner). The following variables were calculated:

a. Energy intake (kcal) during early window = the sum of energy intake before mid-point of eating. Thus, %E intake during early window = ((energy intake (kcal) during early window ÷ total energy intake) × 100). The same calculation method was applied for intake in the late window.
b. For example, carbohydrate intake early window = the sum of carbohydrate intake before the midpoint of eating. Thus, %E from carbohydrate intake during early window = (((carbohydrate intake (g) during early window × 4 kcal) ÷ total energy intake) × 100). The same calculation method was applied for the intake in the late window and suited the other macronutrient (protein and fat) intake.

Night eating syndrome (NES) was characterized by morning anorexia, evening hyperphagia, insomnia and nocturnal ingestions [44]. A night eating questionnaire [44] was used to assess the dietary compliance and change in NES score after the intervention. The total NES score was calculated from 13 items, whereby items 1 to 12 and 14 had a score ranging from 0 to 52. For the scoring system, higher scores indicated greater NES. The Cronbach’s alpha coefficient for the 13 items was 0.61, which indicated moderate internal consistency.

#### 2.3.5. Physical Activity

The validated Malay version of Global Physical Activity Questionnaire (GPAQ) was administered to assess physical activity over the past seven days [45,46]. The metabolic equivalent of task (METs) was used to determine the level of physical activity.

#### 2.3.6. Adiposity, Biochemical and Clinical Parameters

The adiposity parameters included body weight, BMI, body fat percent and waist circumference. Both body weight and body fat percent were determined using a bio-electrical impedance analyzer, TANITA DC-360 (Tanita Corporation of America, Arlington Heights, IL, USA) to the nearest 0.1 kg. Participants were asked to remove their shoes and socks and stand in an upright position with their feet touching the electrodes for the body weight and body fat percent measurement. Height was measured using a portable stadiometer (Seca 213, Hamburg, Germany), and participants were asked to stand in an upright position with their head oriented in the Frankfort horizontal plane during the measurement. BMI was calculated with weight (kg) divided by height squared (m^2^). The waist circumference was measured as the midway between the lower rib margin and iliac crest, to the nearest 0.1 cm using a non-expandable tape.

The biochemical parameters included were fasting blood glucose (FBG), insulin, HbA1c, total cholesterol, high-density lipoprotein-cholesterol (HDL), low-density lipoprotein-cholesterol (LDL-C), triglycerides and uric acid. Homeostatic Model Assessment for Insulin Resistance (HOMA-IR) was calculated from the following formula: fasting blood glucose x insulin level/22.5 [47]. The participants were asked to fast for 8 h (overnight), and blood collection was conducted at 08:00 by a trained phlebotomist. A total of 10 mL of fasting peripheral venous blood was taken for the test. The clinical parameter was the systolic and diastolic blood pressures. The blood pressure was measured using a calibrated digital automatic blood pressure monitor (OMRON, Kyoto, Japan).

### 2.4. Statistical Method

Normality was confirmed using histograms and the skewness and kurtosis levels. Socio-demographic characteristics were analyzed using the chi-square test for categorical variables and independent *t*-test for continuous variables. The pre- and post-intervention changes of the total dietary intake, meal timing, temporal pattern of energy and macronutrient intake, NES score and sleep habits were analyzed using two-way mixed-design ANOVA. All analyses were performed using the intention-to-treat approach. The missing data from the non-completers (*n* = 6) were replaced with their baseline data. Complete case analysis was analyzed and is included in Supplementary Materials as reference. The statistical analyses were conducted using the Statistical Package for Social Science (SPSS, Version 22.0), and the level of significance was set at *p* ≤ 0.05.

## 3. Results

### 3.1. Study Participants

Table 3 includes the socio-demographic information of the participants. A total of 91 non-shift workers participated in this study. Participants were mostly women (74.7%), with an average age of 39.6 years, of Malay ethnicity (97.8%), married (79.1%) with tertiary education (89.0%). About 46 participants were classified as morning chronotype and 45 participants were evening chronotype. There was no significant difference in age, and there was proportionate distribution of gender, race, education level and income between chronotypes. However, evening chronotype had significantly greater proportion of single/divorcee/widow as compared to morning chronotype (*p* = 0.018).

### 3.2. Attendance and Satisfaction Rate

On average, 62 (68.1%) participants attended at least 9 out of the 12 sessions. Of the 91 participants, 85 (93.4%) attended pre- and post-assessments. Two participants dropped out due to conflicting work schedules, and four were lost to follow-up. Looking at the satisfaction rate, 65 (76.4%) participants rated very good, 16 (18.8%) rated good and 4 (4.9%) rated acceptable. Some participants also suggested prolonging the intervention duration (*n* = 11) and more group exercise (*n* = 7).

**Table 3:** Socio-demographic characteristic of participants.

Parameters	Total (*n* = 91)	MT (*n* = 46)	ET (*n* = 45)	*p*-Value
Age ^a^	39.6 ± 6.3	40.8 ± 6.7	38.7 ± 5.8	0.257
Gender ^b^				
Women	68 (74.7)	37 (80.4)	31 (68.9)	0.205
Men	23 (25.3)	9 (19.6)	14 (31.1)	
Race ^b^				
Malay	89 (97.8)	45(97.8)	44 (97.8)	0.987
Chinese	2 (2.2)	1 (2.2)	1 (2.2)	
Marital status ^b^				
Married	72 (79.1)	41 (89.1)	31 (68.9)	**0.018**
Single/divorcee/widow	19 (20.9)	5 (10.9)	14 (31.1)	
Education level ^b^				
Tertiary	81 (89.0)	41 (89.1)	40 (88.9)	0.971
Secondary	10 (11.0)	5 (10.9)	5 (11.1)	
Monthly household income ^b^				
Low	10 (11.0)	5 (10.9)	5 (11.1)	0.136
Middle	61 (67.0)	27 (58.7)	34 (75.6)	
High	20 (22.0)	14 (30.4)	6 (13.3)	
Self-reported medical history				
Hypertension, n (%) ^b^	11 (12.1)	5 (10.9)	6 (13.3)	0.718
Diabetes mellitus, n (%) ^b^	6 (6.6)	2 (4.3)	4 (8.9)	0.434
Dyslipidaemia, n (%) ^b^	11 (12.1)	3 (6.5)	8 (17.8)	0.100

^a^ Data are shown as mean ± standard deviation according to independent *t*-test. ^b^ Data are shown as number (%) according to chi-square test. Bold *p* values indicate statistical significance. Abbreviations: MT, morning chronotype; ET, evening chronotype.

### 3.3. Changes in Dietary, Sleep and Physical Activity between Pre- and Post-Intervention

In terms of total dietary intake, morning and evening chronotypes had significantly reduced the total energy (Mean difference (MD): −471 kcal, 95% confidence interval (CI): −558, −384, *p* < 0.001) and macronutrient intake post-intervention (Table 4). While there was no significant change in % energy from carbohydrate (MD: 0.7%, 95% CI: −0.7, 2.1, *p* = 0.317) pre- and post-intervention in both chronotypes, there was increased % energy from protein (MD: 3.3%, 95% CI: 2.3, 4.2, *p* < 0.001) and reduced % energy from fat sources (MD: −3.9%, 95% CI: −5.3, −2.4, *p* < 0.001). Furthermore, the NES score was significantly improved at post-intervention in both chronotypes (MD: −1.5, 95%CI: −2.5, −0.5, *p* = 0.004). Table 4 presents the outcome for meal timing. Both chronotypes shifted their first and last mealtime earlier, but there was no significant difference between pre- and post-assessments. However, as compared to pre-intervention, both chronotypes displayed a significant earlier midpoint of eating timing during post-intervention, which indicated the eating window had shifted towards the earlier part of the day (MT: 14:02 vs. 13:49; ET: 14:27 vs. 14:18, 95%CI: −0.4, −0.02, *p* = 0.029). Morning chronotypes also consistently displayed significant earlier last mealtime (MD: −0.7 h, 95% CI: −1.2, −0.1, *p* = 0.016) and midpoint of eating (MD: −0.5 h, 95% CI: −0.8, −0.1, *p* = 0.005) during pre- and post-intervention as compared to evening chronotypes. Nonetheless, both chronotypes maintained at least 2.5 h of elapsed time between sleep onset and last meal, even during pre-intervention. Furthermore, morning chronotypes significantly widened the elapse time post-intervention compared to evening chronotype (MD: 0.8 h, 95% CI: 0.1, 1.5, *p* = 0.035).

In terms of sleep habits, both chronotypes significantly increased their sleep duration during work days, but evening chronotypes stood out, with a 1.3 h increase of sleep duration at post-intervention as compared to morning chronotypes (MD: 1.1 h, 95% CI: 0.6, 1.6, *p* < 0.001) (Table 4). However, both chronotypes had a significant decline in sleep duration during free days, particularly among morning chronotypes, who experienced a 0.8 h reduction in sleep (MD: −0.5 h, 95% CI: −0.8, −0.1, *p* = 0.005). Overall, morning and evening chronotypes had an average of 6.6–6.9 h of sleep duration during post-intervention. There was no significant change in average sleep duration within each chronotype (*p* = 0.061); however, evening chronotypes had significantly increased their average sleep duration compared to morning chronotypes (MD: 0.7 h, 95% CI: 0.4, 1.1, *p* < 0.001). Compared to pre-intervention, evening chronotypes had significantly earlier average sleep onset (ET: 23:56 vs. 23:23, 95%CI: −0.4, −0.03, *p* = 0.017) and yet no significant change in average sleep offset (ET: 06:17 vs. 06:18, 95%CI: −0.1, 0.2, *p* = 0.293) during post-intervention. Nonetheless, morning chronotypes still demonstrated a significant earlier sleep onset and sleep offset trend compared to evening chronotypes during pre- and post-intervention. Evening chronotypes had greater social jetlag than morning chronotypes during pre- and post-intervention. However, evening chronotypes also had greater reduction in social jetlag compared to morning chronotypes after the intervention (MD: 19 min, 95% CI: 1.7, 36.3, *p* = 0.031). Both chronotypes had also improved their physical activity level following the intervention (MD: 1700.0, 95% CI: 1150.0, 2250.0, *p* < 0.001).

### 3.4. Changes in Temporal Pattern of Energy and Macronutrient Intake between Pre- and Post-Intervention

**Figure 2:**
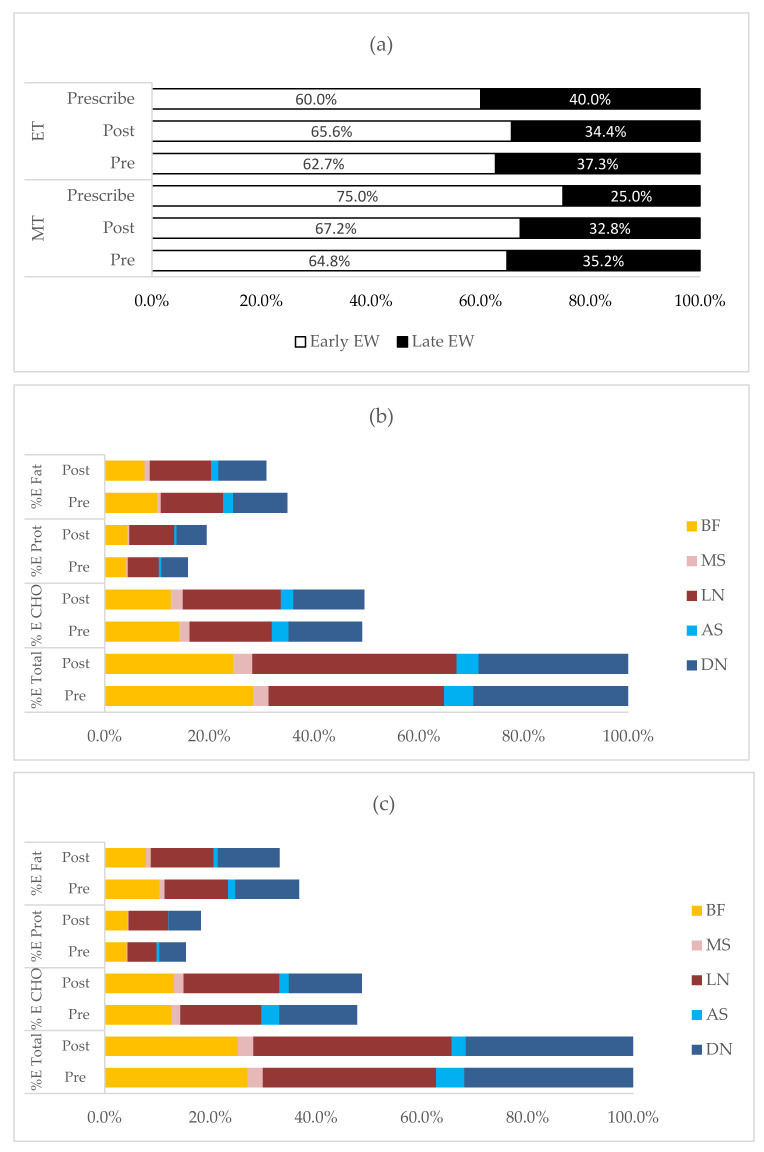
Pre–post differences in temporal patterns of energy and macronutrient intake based on early and late window. (**a**) The distribution of % E intake during pre- and post-intervention according to morning and evening chronotypes. (**b**) The distribution of % E from CHO, protein and fat based on early (breakfast, morning snack and lunch) and late (afternoon snack and dinner) windows in MT. (**c**) The distribution of % E from CHO, protein and fat based on early (breakfast, morning snack and lunch) and late (afternoon snack and dinner) windows in ET. Detailed statistical values described in Supplementary Table S2a,b. Abbreviations: E, energy; CHO, carbohydrate; prot, protein; MT, morning chronotype; ET, evening chronotype; EW, eating window; % E, percentage energy; BF, breakfast; MS, morning snack; LN, lunch; AS, afternoon snack; DN, dinner.

Figure 2a reveals that there was a significant increase in the % energy intake during the early window, coupled with a decreased intake in the late window among morning and evening chronotypes, following the intervention. Though morning chronotypes did not achieve the recommended intake (75% during early window and 25% late window), they had greater % in energy intake during the early window during post-intervention as compared to pre-intervention (64.8 vs. 67.2%, MD: 2.8, 95%CI: 0.3, 5.1, *p* = 0.028), and vice versa for the intake in the late window. The pre-intervention intake of evening chronotypes already met the recommended intake (60% during early window and 40% during late window). Post-intervention, evening chronotypes had further increased their % energy intake during early window (62.7 vs. 65.6%, MD: 2.8, 95%CI: 0.3, 5.1, *p* = 0.028).

Figure 2b,c further details the % energy intake according to breakfast, morning snack, lunch, afternoon snack and dinner. Both chronotypes had reduced % energy intake during breakfast after the intervention (MT: 28.3 vs. 24.5%; ET: 27.0 vs. 25.2%, MD: −2.8, 95% CI: −4.8, −0.9, *p* = 0.004), which resulted in a significant reduction in energy from fat sources (MT: 10.0 vs. 7.6%; ET: 10.3 vs. 7.8%, MD: −2.5, 95% CI: −3.4, −1.5, *p* < 0.001). There were no significant changes during morning snack (MT: 3.0 vs. 3.7%; ET: 2.9 vs. 2.9%, MD: 0.4, 95% CI: −1.0, 1.7, *p* = 0.593). Both chronotypes had greater % energy intake during lunch post-intervention (MT: 33.5 vs. 39.0%; ET: 32.8 vs. 37.5%, MD: 5.2, 95% CI: 2.7, 7.6, *p* < 0.001), which was mainly from carbohydrate sources (MT: 15.7 vs. 18.7%; ET: 15.3 vs. 18.1%, MD: 2.9, 95% CI: 1.4, 4.4, *p* < 0.001) and protein sources (MT: 5.9 vs. 8.6%; ET: 5.5 vs. 7.5%, MD: 2.3, 95% CI: 1.7, 3.0, *p* < 0.001). For afternoon snack, both chronotypes had reduced their intake as compared to pre-intervention (MT: 5.6 vs. 4.2%; ET: 5.3 vs. 2.7%, MD: −1.9, 95% CI: −3.6, −0.1, *p* = 0.035), which resulted from a reduction in carbohydrate sources (MT: 3.2 vs. 2.4%; ET: 3.4 vs. 1.8%, MD: −1.2, 95% CI: −2.2, −0.2, *p* = 0.021) and fat sources (MT: 1.9 vs. 1.4%; ET: 1.4 vs. 0.8%, MD: −0.5, 95% CI: −1.2, 0.1, *p* = 0.092). For dinner, there was a reduction in energy intake, but there was no significant difference between pre- and post-intervention (MT: 29.6 vs. 28.6%; ET: 32.0 vs. 31.7%, MD: −0.7, 95% CI: −3.1, 1.8, *p* = 0.594). Both chronotypes had a reduction in % energy intake from carbohydrate sources (MT: 14.1 vs. 13.6%; ET: 14.8 vs. 13.9%, MD: −0.7, 95% CI: −2.0, 0.6, *p* = 0.279) and fat sources (MT: 10.4 vs. 9.2%; ET: 12.1 vs. 11.7%, MD: −0.7, 95% CI: −2.0, 0.5, *p* = 0.248) and a significant increase in energy from protein sources (MT: 5.1 vs. 5.8%; ET: 5.1 vs. 6.1%, MD: 0.9, 95% CI: 0.3, 1.4, *p* = 0.002).

### 3.5. Changes in Adiposity and Biochemical Parameters between Pre- and Post-Intervention

In overall, participants lost 4.8% from baseline weight (−4.0 ± 4.4 kg/m^2^). Both chronotypes had a significant reduction in all adiposity parameters after 12 weeks of the integrated chrono-nutrition weight reduction program (Table 5). Morning chronotypes lost 5.3 % weight from the baseline weight, with a reduction of −1.7 kg/m^2^ in BMI, −2.2 % body fat and −3.6 cm of waist circumference (*p* < 0.001). Evening chronotypes lost 4.3% body weight, with a reduction of −1.4 kg/m^2^ in BMI, −1.8 % body fat and −4.1 cm of waist circumference (*p* < 0.001). Nonetheless, there were no significant differences observed between the two groups in all adiposity parameters.

In terms of biochemical parameters, both chronotypes had significant reduction in insulin (MD: −3.2, 95% CI: −4.5, −1.8, *p* < 0.001), insulin resistance marker-HOMA-IR [MD: −0.7, 95% CI: −1.1, −0.4, *p* < 0.001], triglyceride (MD: −0.1, 95% CI: −0.2, −0.01, *p* = 0.029), HDL-C (MD: −0.04, 95% CI: −0.1, −0.01, *p* = 0.024) and uric acid level (MD: −0.01, 95% CI: −0.02, −0.001, *p* = 0.028). For blood pressure, both chronotypes had significant reduction in systolic (MD: −8.5, 95% CI: −10.7, −6.2, *p* < 0.001) and diastolic (MD: −4.3, 95% CI: −6.7, −2.0, *p* < 0.001) blood pressure post-intervention. Despite this, there was no significant differences observed between chronotypes in all biochemical parameters and blood pressures.

### 3.6. Refinement of the Integrated Chrono-Nutrition Weight Reduction Intervention

After the feasibility study, it became apparent that there were several considerations and changes required to tailor the intervention to the needs of overweight/obese morning and evening chronotypes to enhance the successfulness of the implementation and improve the adherence towards the recommendations. Table 6 consists of refinement to the chrono-nutrition components, which focused on the temporal pattern energy intake domain. Our study results suggested that both chronotypes are capable of eating more during the earlier part of the day and eating less later. Thus, there is no obligatory or different cut-off for the temporal energy intake recommendation. In addition, the majority of morning chronotypes did not achieve the 75% energy intake recommendation in the early window. Our recent work shows that the energy intake of 62–64% during the early window has benefits for metabolic health among overweight/obese adults [48]. Therefore, the recommendation was revised to improve compliance and at the same time achieve a significant level of intake towards clinical benefits. Another refinement was needed for the food menu. Initially, we had provided the meal plan using food groups to give participants freedom in menu planning. However, the participants reported that they preferred a complete set of menus, including the dishes to eat throughout the intervention period.

In demonstrating feasibility through attendance, 68% of the participants attended at least 9 out of the 12 weeks sessions. This workplace weight reduction program was conducted during working hours, close to participant’s workplace. In addition, participants were required to obtain permission from their employer to participate in this program. However, the most frequent reasons for being absent were scheduling conflicts, such as a sudden change in work schedule due to meetings. Nonetheless, 93% of the participants were present during post-intervention assessments. The timing of the program should be revised with the participants prior to the program (for example, to be held after working hours) to enhance the participation rate.

## 4. Discussions

The integrated chrono-nutrition weight reduction program was designed as an adjunct strategy to a hypocaloric diet and physical activity in traditional weight reduction interventions. Participants completing this program had successfully improved their chrono-nutrition practice by consuming a greater food intake during earlier part of the day coupled with smaller intake during later part of the day in both morning and evening chronotypes. Participants also had improved their meal timing, NES score and sleep habits following the intervention. Moreover, the current program had a lower attrition rate as compared to our previous weight reduction program (6.6 vs. 41.5%) [38].

Following the intervention, both chronotypes had a small but significant increase of energy intake during the early window as compared to pre-intervention (2.4–2.9%). It has been reported that evening chronotypes are linked with lower morning energy intake and greater intake at night [34]. Thus, evening chronotypes were given a lower energy intake recommendation for the early window as compared to morning chronotypes, and vice versa for late window. However, our baseline data showed that evening chronotypes had greater energy intake during the early window, similar to morning chronotypes (62.7 vs. 64.8%, *p* = 0.308). Thus, evening chronotypes easily exceeded the recommended intake for at least 60% of the energy intake during early window. These results implied that the recommendation for a greater energy intake during the earlier part and smaller energy intake during the latter part of the day is practical/manageable for evening chronotypes. Furthermore, evening chronotypes’ mealtime in this study was constrained to fixed-day working hours (starting from 07:30–08:30 to 16:30–17:30), which resulted in two of the main meals (breakfast and lunch) occurring during working time. The greater food intake among evening chronotypes during the early window might have been influenced by the energy demand for work performance and social occasions. Even though morning chronotypes did not achieve the recommended intake for at least 75% of energy intake during the early window, they had significantly increased their early window intake as compared to pre-intervention. One of the possible reasons for this result is the reduction in energy intake during breakfast, which was also seen in the evening chronotype. Possibly, the participants had improved their food choices during breakfast, which resulted in a significant reduction of energy, carbohydrates and fat (Figure 2b,c). A significant reduction in eating out frequency (food away from home) during breakfast was observed among all participants (4.2 times/week vs. 2.3 times/week, *p* < 0.001: Supplementary Table S4). This reduction in the frequency of eating out could reduce the calorie intake, as outside food is mostly calorie-dense and high in sodium and fat [49]. The greatest energy intake during the early window was contributed from lunch, for which both chronotypes had greater carbohydrate and protein intake and yet no changes in fat intake as compared to pre-intervention. Therefore, the recommendations on the early midpoint of eating or main meal are crucial to avoid more calorie deposits towards the later part of the day.

Our participants also consumed the most calories during lunch, which could lead to lower calorie intake during the next meal. The reduction in energy intake during the late window was contributed from the afternoon snack. Possibly, our participants had improved their food choices for afternoon snacks to healthier food choices, which led to a reduction in calories from carbohydrates and fat. A recent weight intervention study demonstrated that participants that lost more weight had healthier food choices [50]. As for dinner, there was no notable reduction in energy intake as compared to pre-intervention among both chronotypes. Despite this, morning and evening chronotypes had earlier last mealtimes as compared to pre-intervention, and they also maintained the 2.5 h gap between last meal and sleep onset, which is also an essential aspect of chrono-nutrition. Overall, both chronotypes managed to increase their consumption during the early window and reduce it during the late window. There seems to be a definite need for a future study to examine the differences in food choices between pre-and post-dietary intervention in relation to chronotypes, which could be targeted to sustain the weight loss effect.

In terms of meal timing, evening chronotypes had later mealtime as compared to morning chronotypes during pre- and post-intervention. However, there was no significant difference in first mealtime between morning and evening chronotypes among our sample population. Possibly, the first mealtime was influenced more by work schedule than the last mealtime. Nonetheless, our study found that both chronotypes had a midpoint earlier than 15:00 for eating since pre-intervention. They also had further shifted the midpoint of eating earlier by having earlier first and last mealtime after the intervention. This is possibly due to participants having fixed working hours, and thus the participants had better control of their mealtime. Furthermore, all our participants lived within the same area of their workplace. This factor reduced the time spent for transportation to commute from the workplace to home, which could result in a delay in dinner timing. Morning and evening chronotypes also maintained the 3 h elapsed time between the last mealtime and sleep onset following the intervention. Melatonin onset starts within 2–3 h before the usual sleep onset [51]. It has been demonstrated that the simultaneous increase in melatonin and glucose level impairs glucose tolerance because melatonin inhibits glucose-stimulated insulin secretion [52]. In addition, greater energy intake closer to melatonin onset is related to a greater body fat percentage [53]. Therefore, the recommendations on meal timing are practical for our participants, regardless of their chronotypes, and should be continued.

Interestingly, our results suggested that the sleep recommendations had benefited evening chronotypes more than morning chronotypes. This is because evening chronotypes had shorter sleep duration than morning chronotypes at pre-intervention. After the intervention, evening chronotypes had increased their sleep duration during work days to more than 7 h daily, thus reducing the discrepancy between work and free days. This discrepancy is known as social jetlag, which is the misalignment between biological clock timing and social/work timing [54]. Evening chronotypes are more susceptible to greater social jetlag than morning chronotypes because the sleep–wake timing is a constraint to early work schedules and results in accumulated sleep debt during work days [55]. Thus, evening chronotypes showed greater reduction in social jetlag than morning chronotypes following the intervention. Unexpectedly, morning chronotypes demonstrated a non-significant reduction in average sleep duration. Nonetheless, both chronotypes had more than 6 h of sleep daily. A 12-month follow-up study post-lifestyle intervention reported those who slept less than 6 h/day had a smaller reduction in waist circumference compared to those who slept more than 7 h/day [56]. Sleep and chrono-nutrition is interrelated, as it was shown that short sleepers were associated with longer eating windows [57], which could lead to greater food intake [58]. Despite this, both chronotypes demonstrated a reduction in total eating window post-intervention. A recent large cross-sectional study among working adults in Malaysia discovered that more than 50% of the respondents sleep less than 7 h/day, which was associated with having children, lifestyle factors, poor sleeping conditions and mental health issues [59]. Hence, strategies to enhance sleep duration might involve education on the importance of adequate sleep and addressing the abovementioned factors.

Generally, both chronotypes achieved a modest but significant weight loss following the weight reduction program, and there was no significant difference in all adiposity parameters between morning and evening chronotypes. This finding might suggest that a combination of hypocaloric diet, physical activity and improvement in chrono-nutrition is beneficial for a modest weight reduction in both chronotypes. Nonetheless, it is important to also note that earlier works suggested that morning chronotypes maintained a greater weight loss compared to evening chronotypes during one-year follow-up [60] and even after 6 years post-bariatric surgery [24]. The greater weight loss maintainers were characterized by longer sleep duration and better sleep quality [60] and earlier mealtime [24]. In our study, both chronotypes had significantly improved their midpoint of eating post-intervention, and yet morning chronotypes still had earlier midpoints of eating compared to evening chronotypes. Even though there was a significant difference in mealtime between morning and evening chronotypes, our current study implies that the difference is negligible for short-term weight reduction, as both chronotypes achieved a satisfactory weight loss. Despite this, more controlled, long-term experimental studies are required to examine the weight loss progress in each chronotype and identify the possible differentiating factors.

This is a distinctive study incorporating a multidomain of chrono-nutrition in a single weight reduction intervention as an adjunct strategy to calorie restriction and physical activity. Furthermore, the changes in chrono-nutrition practiced, such as temporal eating pattern, meal timing and NES score, were described in detail. A number of limitations are acknowledged and should be considered in interpreting the current findings. This is a feasibility study, which provides only the preliminary evidence on the acceptance of an integrated chrono-nutrition weight reduction program among non-shift workers. Second, the chronotype was categorized based on the median sample population midpoint of sleep instead of using cut-off values reported by other countries [61]. This could result in fewer extreme morning and evening chronotypes and thus reduce meaningful differences between chronotypes. Third, most of the participants are of Malay ethnicity, because this study was conducted close to government offices that employ predominantly non-shift workers of Malay ethnicity. Thus, the finding might not precisely represent the general multi-ethnic Malaysian population, and the differences in ethnic dietary patterns may be reduced.

## 5. Conclusions

These findings demonstrated that an integrated chrono-nutrition weight reduction program improved participants’ temporal patterns of energy intake, meal timing, night eating syndrome scores and sleep habits among morning and evening chronotypes in overweight/obese non-shift workers. The current program shows a high acceptance and low attrition rate. The uncertainties on the key intervention’s domain, which is the temporal pattern of energy recommendation, were addressed and revised to tailor the intervention to the target population. Future study is needed to investigate the effectiveness of the revised module in improving weight loss, metabolic status and lifestyle behaviors, including dietary and sleep patterns.

## Figures and Tables

**Table 1:** Proposed recommendations for chrono-nutrition components.

Components	Recommendation	Reference
Temporal pattern of energy intake	MT: BF—30%, MS—10%, LN—35%, AS—5% and DN—20% ET: BF—25%, MS—5%, LN—35%, AS—10% and DN—30%.	[26]
Mealtime	To eat main meal before 15:00. To eat dinner at least 2 ½ h before sleep onset. To avoid night eating.	[35]
Sleep	Sleep hours: ● Minimum: 6 h ● Optimum: 7–9 h	[36]

Abbreviation: MT, morning chronotype; ET, evening chronotype; BF, breakfast; MS, morning snack; LN, lunch; AS, afternoon snack; DN, dinner.

**Table 2:** Comparison between the previous SLIMSHAPE™ and SLIMSHAPE™ Chrono.

SLIMSHAPE™	SLIMSHAPE™ Chrono
**Similarities**	
**Dietary** Calorie prescription: *Men: 1600–1800 kcal/day* *Women: 1200–1500 kcal/day* Macronutrient distribution: 50% CHO, 20% protein and 20% fat. Meal plan guide. Healthy eating guide through calorie counting, healthy cooking method and demonstration, understanding food labels, identify sugar-sweetened beverages and fats in cooked food.	
**Physical activity and exercise** Weekly group exercise lead by exercise physiologist, including resistance tube exercise, aerobic exercise, high-intensity interval training and yoga. Encouragement to be physically active.	
**Behavioral therapy** Group social support. Weekly weight monitoring. Session with psychologist on identifying barriers in weight reduction journey and motivational talk delivered by successful weight loss maintainers.	
**Differences**	
**Duration** 16 weeks/weekly session 2 h each session	**Duration** 12 weeks/weekly session 2-1/2 h each session
**Chronotype** Not assessed.	**Chronotype** Assessed.
**Temporal pattern of energy intake** No recommendation.	**Temporal pattern of energy intake** Prescription according to chronotype **MT**: Early window: 75% EI *(BF: 30%, MS: 10%, LN: 35%)* Late window: 25% EI *(AS: 5% and DN: 20%)* **ET**: Early window: 60% EI *(BF: 20%, MS: 5%, LN: 35%)* Late window: 40% EI *(AS: 10% and DN: 30%)*
**Mealtime** No recommendation.	**Mealtime** Midpoint of eating before 15:00. Elapsed time between last meal and sleep onset ≥ 2-1/2 h. To avoid/reduce night eating.
**Sleep** No recommendation.	**Sleep** Sleep hours: *Minimum: 6 h* *Optimum: 7–9 h* To maintain regular sleep–wake timing

Abbreviation: CHO, carbohydrate; MT, morning chronotype; ET, evening chronotype; EI, energy intake; BF, breakfast; MS, morning snack; LN, lunch; AS, afternoon snack; DN, dinner.

**Table 4:** Pre–post intervention differences in total dietary intake, meal timing, NES score, sleep habits and physical activity.

	MT (*n* = 46)		ET (*n* = 45)		Mean Difference (95% CI)		
	Pre	Post	Pre	Post	Time	CT	Time × CT
Total dietary intake							
EI (kcal/day)	1660 ± 445	1228 ± 326	1878 ± 466	1373 ± 407	−471 (−558, −384) ***	−180 (−330, −32) *	68 (−106, 242)
CHO (g/day)	204.0 ± 64.5	152.1 ± 45.1	224.0 ± 65.3	167.1 ± 52.5	−55.3 (−67.5, −43.0) ***	−17.4 (−3.1, 37.9)	6.5 (−18.1, 31.1)
% E CHO	49.2 ± 5.8	49.5 ± 6.3	47.7 ± 5.7	48.7 ± 6.0	0.7 (−0.7, 2.1)	1.1 (−1.0, 3.0)	−0.4 (−3.2, 2.5)
Protein (g/day)	66.0 ± 16.8	59.8 ± 11.3	72.8 ± 16.9	62.5 ± 16.9	−8.6 (−12.0, −5.3) ***	−4.9 (−10.5, 0.7)	3.94 (−2.8, 10.6)
% E protein	15.9 ± 3.3	19.5 ± 3.8	15.5 ± 2.0	18.2 ± 4.1	3.3 (2.3, 4.2) ***	0.9 (−0.2, 1.9)	0.9 (−1.0, 2.8)
Fat (g/day)	64.4 ± 19.4	42.3 ± 16.6	76.9 ± 22.5	50.5 ± 20.4	−23.7 (−28.3, −19.1) ***	−10.3 (−17.2, −3.4) *	3.29 (−5.9, 12.5)
% E fat	34.9 ± 4.9	31.0 ± 6.2	36.8 ± 5.0	33.1 ± 6.3	−3.9 (−5.3, −2.4) ***	−2.1 (−3.9, −0.2) *	−0.3 (−3.2, 2.6)
Meal timing							
First meal (hh:mm)	08:12 ± 0:40	08:04 ± 0:46	08:24 ± 0:43	08:22 ± 0:43	−0.1 (−0.2, 0.03)	−0.3 (−0.5, 0.03)	0.1 (−0.3, 0.1)
Last meal (hh:mm)	19:52 ± 1:16	19:33 ± 1:08	20:29 ± 1:45	20:14 ± 1:37	−0.3 (−0.6, 0.04)	−0.7 (−1.2, −0.1) *	−0.1 (−0.7, 0.6)
Total eating window (h)	11.7 ± 1.6	11.5 ± 1.4	12.1 ± 1.7	11.9 ± 1.6	−0.2 (−0.6, 0.2)	−0.4 (−1.0, 0.2)	0.0 (−0.7, 0.7)
Midpoint eating (hh:mm)	14:02 ± 0:38	13:49 ± 0:42	14:27 ± 1:01	14:18 ± 0:57	−0.2 (−0.4, −0.02) *	−0.5 (−0.8, −0.1) **	−0.1 (−0.4, 0.2)
Elapse SOn-last meal (h)	3.1 ± 1.5	3.5 ± 1.3	3.5 ± 1.8	3.2 ± 1.6	0.1 (−0.3, 0.4)	0.0 (−0.5, 0.5)	0.8 (0.1, 1.5) *
NES score	10.0 ± 5.1	8.9 ±4.3	10.7 ±5.4	8.9 ± 5.0	−1.5 (−2.5, −0.5) **	−0.4 (−2.2, 1.5)	0.7 (−1.3, 2.7)
Sleep							
Work SD (hour)	6.6 ± 1.0	6.7 ± 0.9	6.1 ± 0.9	7.4 ± 1.2	0.7 (0.4, 1.0) ***	−0.1 (−0.4, 0.2)	−1.1 (−1.6, −0.6) ***
Free SD (hour)	7.3 ± 1.2	6.5 ± 0.9	6.8 ± 1.5	6.7 ± 0.8	−0.5 (−0.8, −0.1) ***	0.2 (−0.2, 0.5)	−0.7 (−1.3, −0.1) *
Average SOn (hh:mm)	22:57 ± 0:48	23:05 ± 0:50	23:56 ± 0:53	23:23 ± 0:57	−0.2 (−0.4, −0.03) *	−0.6 (−1.0, −0.3) ***	0.7 (0.3, 1.0) ***
Average SOff (hh:mm)	05:46 ± 0:31	05:38 ± 0:29	06:17 ± 0:41	06:18 ± 0:47	−0.1 (−0.1, 0.2)	−0.6 (−0.8, −0.4) ***	−0.2 (−0.4, 0.1)
Average SD (hour)	6.8 ± 0.9	6.6 ± 0.8	6.3 ± 0.9	6.9 ± 0.7	0.2 (−0.0, 0.4)	0.1 (−0.2, 0.4)	−0.8 (−1.2, −0.4) ***
Social jetlag (min)	18.2 ± 30.0	23.6 ± 23.6	53.1 ± 45.0	39.5 ± 42.6	−4.1 (−12.7, 4.6)	−25.4 (−37.8, −13.0) ***	19.0 (1.7, 36.3) *
Physical activity (MET)	1693.7 ± 303.8	3509.4 ± 387.0	1611.5 ± 303.8	3462.3 ± 387.0	1700.0 (1150.0, 2250.0) ***	−124.9 (−865.4, 615.5)	48.1 (−1051.9, 1148.1)

Data are presented in mean and standard deviation. Statistical significance using two-way mixed ANOVA test at * *p* < 0.05, ** *p* < 0.01 and *** *p* < 0.001. Abbreviations: MT, morning chronotype; ET, evening chronotype; CI, confidence interval; CT, chronotype; EI, energy intake; CHO, carbohydrate; % E, percentage energy; hh:mm, local time in hour and minute; h, hour; min, minute; Elapse SOn-last meal, elapse time between last mealtime and sleep onset; NES, night eating syndrome; SD, sleep duration. Refer to Supplementary Table S1 for the complete case analysis.

**Table 5:** Pre–post intervention differences in adiposity, and biochemical parameters.

	MT (*n* = 46)		ET (*n* = 45)		Mean Difference (95% CI)	
	Pre	Post	Pre	Post	Time	Time × CT
Adiposity						
Weight (kg)	79.8 ± 16.4	75.6 ± 15.6	81.5 ± 14.1	77.7 ± 13.2	−4.0 (−4.9, −3.1) ***	−0.5 (−2.4, 1.3)
BMI (kg/m^2^)	31.3 ± 4.3	29.6 ± 4.5	31.2 ± 4.7	29.8 ± 4.7	−1.5 (−1.9, −1.2) ***	−0.2 (−0.9, 0.5)
Body fat (%)	41.1 ± 7.4	39.0 ± 8.5	39.7 ± 8.1	37.8 ± 8.5	−2.0 (−2.5, −1.4) ***	−0.3 (−1.4, 0.8)
WC (cm)	94.0 ± 11.8	90.3 ± 11.7	94.1 ± 10.1	90.0 ± 9.7	−3.9 (−4.9, −2.9) ***	0.4 (−1.6, 2.5)
Biochemical						
FBG (mmol/L)	5.0 ± 0.5	4.9 ± 0.6	5.0 ± 0.7	5.0 ± 0.7	−0.1 (−0.2, 0.03)	−0.01 (−0.2, 0.2)
Insulin (μIU/mL)	13.9 ± 7.4	10.3 ± 6.7	12.7 ± 9.3	9.9 ± 5.8	−3.2 (−4.5, −1.8) ***	−0.8 (−3.5, 1.9)
HbA1c (%)	5.8 ± 0.5	5.8 ± 0.7	5.8 ± 0.6	5.8 ± 0.5	0.01 (−0.1, 0.1)	0.1 (−0.1, 0.2)
HOMA-IR	3.1 ± 1.8	2.3 ± 2.0	2.9 ± 2.4	2.3 ± 1.6	−0.7 (−1.1, −0.4) ***	−0.1 (−0.8, 0.6)
TC (mmol/L)	5.2 ± 1.0	5.1 ± 1.1	5.1 ± 0.9	5.0 ± 0.8	−0.1 (−0.2, 0.1)	0.1 (−0.2, 0.4)
TG (mmol/L)	1.3 ± 0.8	1.2 ± 0.7	1.4 ± 0.8	1.3 ± 0.9	−0.1 (−0.2, −0.01) *	−0.01 (−0.2, 0.2)
LDL-C (mmol/L)	3.3 ± 0.94	3.3 ± 1.0	3.1 ± 0.9	3.1 ± 0.8	0.04 (−0.1, 0.2)	0.1 (−0.2, 0.3)
HDL-C (mmol/L)	1.3 ± 0.3	1.3 ± 0.3	1.4 ± 0.3	1.3 ± 0.3	−0.04 (−0.1, −0.01) *	0.03 (−0.1, 0.1)
Uric acid (mmol/L)	0.4 ± 0.1	0.3 ± 0.1	0.4 ± 0.1	0.4 ± 0.1	−0.01 (−0.03, −0.00) *	0.00 (−0.02, 0.02)
Blood pressure						
Systolic (mmHg)	128.5 ± 10.8	118.9 ± 12.2	135.6 ± 12.0	128.2 ± 14.2	−8.5 (−10.7, −6.2) ***	−2.2 (−6.7, 2.3)
Diastolic (mmHg)	80.4 ± 9.6	75.3 ± 12.4	81.4 ± 9.0	77.7 ± 9.6	−4.3 (−6.7, −2.0) ***	−1.4 (−6.0, 3.2)

Data are presented in mean and standard deviation. Statistical significance using two-way mixed ANOVA test at * *p* < 0.05, ** *p* < 0.01 and *** *p* < 0.001. Abbreviations: MT, morning chronotype; ET, evening chronotype; CI, confidence interval; CT, chronotype; BMI, body mass index; WC, waist circumference; FBG, fasting blood glucose; HOMA-IR, homeo-static model assessment of insulin resistance; TC, total cholesterol; LDL-C, low-density lipoprotein cholesterol; HDL-C, high-density lipoprotein cholesterol. Refer to Supplementary Table S3 for the complete case analysis.

**Table 6:** Refinement in chrono-nutrition components based on the feasibility study.

Components	Refinement in Interventions	Rationale
Temporal pattern of energy intake	Temporal energy prescription is the same for morning and evening chronotypes: Early window: 65–70% EI *BF: 25–30%* *MS: 0–5%* *LN: 40%* Late window: 30–35% EI *AS: 0–5%* *DN: 25–30%* Provide a complete set of menus, including the dishes for 12 weeks (intervention period).	There was no significant difference in % energy intake during early and late window between morning and evening chronotypes during pre- and post-intervention. Greater energy intake towards earlier part of the day and smaller intake towards later part of the day is beneficial for both chronotypes [48].
Meal timing	No changes in meal timing recommendations.	The participants adapt well to the recommendation.
Sleep	No changes in sleep hours recommendation. Increase the sleep session to improve understanding on the importance of adequate sleep.	Morning chronotypes had reduced sleep duration on free days.

Abbreviations: EI, energy intake; BF, breakfast; MS, morning snack; LN, lunch; AS, afternoon snack; DN, dinner.

## Data Availability

The data presented in this study are part of ongoing doctoral research of F.H.M. Hence, the data cannot be publicly released. However, data are available upon request from the corresponding author (Z.A.M.).

## Supplementary Materials

The following supporting information can be downloaded at: https://www.mdpi.com/article/10.3390/ijerph19084469/s1, Table S1: Pre-post intervention differences in total dietary intake, meal timing, NES score, sleep habits and physical activity (Complete case, *n* = 85); Table S2: (a): Distribution of % total energy and energy from macronutrients in early and late windows; (b): Distribution of % total energy and energy from macronutrients in early and late window (Complete case, *n* = 85); Table S3: Pre-post intervention differences in adiposity, and biochemical parameters (Complete case, *n* = 85). Table S4: Eating out (food-away from home) frequency in a week.

## References

[1] Fruh S.M. (2017). Obesity: Risk factors, complications, and strategies for sustainable long-term weight management. J. Am. Assoc. Nurse Pract..

[2] Lin X., Xu Y., Xu J., Pan X., Song X., Shan L., Zhao Y., Shan P.F. (2020). Global burden of noncommunicable disease attributable to high body mass index in 195 countries and territories, 1990–2017. Endocrine.

[3] Kompaniyets L., Goodman A.B., Belay B., Freedman D.S., Sucosky M.S., Lange S.J., Gundlapalli A.V., Boehmer T.K., Blanck H.M. (2021). Body Mass Index and Risk for COVID-19–Related Hospitalization, Intensive Care Unit Admission, Invasive Mechanical Ventilation, and Death—United States, March–December 2020. MMWR Surveill. Summ..

[4] Tremmel M., Gerdtham U.G., Nilsson P.M., Saha S. (2017). Economic burden of obesity: A systematic literature review. Int. J. Environ. Res. Public Health.

[5] Peñalvo J.L., Sagastume D., Mertens E., Uzhova I., Smith J., Wu J.H.Y., Bishop E., Onopa J., Shi P., Micha R. (2021). Effectiveness of workplace wellness programmes for dietary habits, overweight, and cardiometabolic health: A systematic review and meta-analysis. Lancet Public Health.

[6] Courcoulas A.P., Christian N.J., Belle S.H., Berk P.D., Flum D.R., Garcia L., Horlick M., Kalarchian M.A., King W.C., Mitchell J.E. (2013). Weight change and health outcomes at 3 years after bariatric surgery among individuals with severe obesity. JAMA.

[7] Khera R., Murad M.H., Chandar A.K., Dulai P.S., Wang Z., Prokop L.J., Loomba R., Camilleri M., Singh S. (2016). Association of Pharmacological Treatments for Obesity With Weight Loss and Adverse Events: A Systematic Review and Meta-analysis. JAMA.

[8] Galasso L., Montaruli A., Jankowski K.S., Bruno E., Castelli L., Mulè A., Chiorazzo M., Ricceri A., Erzegovesi S., Caumo A. (2020). Binge eating disorder: What is the role of physical activity associated with dietary and psychological treatment?. Nutrients.

[9] Romieu I., Dossus L., Barquera S., Blottière H.M., Franks P.W., Gunter M., Hwalla N., Hursting S.D., Leitzmann M., Margetts B. (2017). Energy balance and obesity: What are the main drivers?. Cancer Causes Control.

[10] Almoosawi S., Vingeliene S., Karagounis L.G., Pot G.K. (2016). Chrono-nutrition: A review of current evidence from observational studies on global trends in time-of-day of energy intake and its association with obesity. Proc. Nutr. Soc..

[11] Pot G.K. (2021). Chrono-nutrition—An emerging, modifiable risk factor for chronic disease?. Nutr. Bull..

[12] Makarem N., Sears D.D., St-Onge M.P., Zuraikat F.M., Gallo L.C., Talavera G.A., Castaneda S.F., Lai Y., Mi J., Aggarwal B. (2020). Habitual nightly fasting duration, eating timing, and eating frequency are associated with cardiometabolic risk in women. Nutrients.

[13] Thomas E.A., Zaman A., Cornier M.A., Catenacci V.A., Tussey E.J., Grau L., Arbet J., Broussard J.L., Rynders C.A. (2021). Later meal and sleep timing predicts higher percent body fat. Nutrients.

[14] Dashti H.S., Gómez-Abellán P., Qian J., Esteban A., Morales E., Scheer F.A.J.L., Garaulet M. (2021). Late eating is associated with cardiometabolic risk traits, obesogenic behaviors, and impaired weight loss. Am. J. Clin. Nutr..

[15] Madjd A., Taylor M.A., Delavari A., Malekzadeh R., MacDonald I.A., Farshchi H.R. (2016). Beneficial effect of high energy intake at lunch rather than dinner on weight loss in healthy obese women in a weight-loss program: A randomized clinical trial. Am. J. Clin. Nutr..

[16] Jakubowicz D., Barnea M., Wainstein J., Froy O. (2013). High Caloric intake at breakfast vs. dinner differentially influences weight loss of overweight and obese women. Obesity.

[17] Raynor H.A., Li F., Cardoso C. (2018). Daily pattern of energy distribution and weight loss. Physiol. Behav..

[18] Xiao Q., Garaulet M., Scheer F.A.J.L. (2019). Meal timing and obesity: Interactions with macronutrient intake and chronotype. Int. J. Obes..

[19] Vera B., Dashti H.S., Gómez-Abellán P., Hernández-Martínez A.M., Esteban A., Scheer F.A.J.L., Saxena R., Garaulet M. (2018). Modifiable lifestyle behaviors, but not a genetic risk score, associate with metabolic syndrome in evening chronotypes. Sci. Rep..

[20] Bazzani A., Marantonio S., Andreozzi G., Lorenzoni V., Bruno S., Cruz-Sanabria F., d’Ascanio P., Turchetti G., Faraguna U. (2022). Late chronotypes, late mealtimes. Chrononutrition and sleep habits during the COVID-19 lockdown in Italy. Appetite.

[21] Maukonen M., Kanerva N., Partonen T., Kronholm E., Tapanainen H., Kontto J., Männistö S. (2017). Chronotype differences in timing of energy and macronutrient intakes: A population-based study in adults. Obesity.

[22] Fárková E., Šmotek M., Bendová Z., Manková D., Kopřivová J. (2021). Chronotype and social jet-lag in relation to body weight, apetite, sleep quality and fatigue. Biol. Rhythm Res..

[23] Muscogiuri G., Barrea L., Aprano S., Framondi L., Di Matteo R., Laudisio D., Pugliese G., Savastano S., Colao A. (2020). Chronotype and adherence to the mediterranean diet in obesity: Results from the opera prevention project. Nutrients.

[24] Ruiz-Lozano T., Vidal J., De Hollanda A., Canteras M., Garaulet M., Izquierdo-Pulido M. (2016). Evening chronotype associates with obesity in severely obese subjects: Interaction with CLOCK 3111T/C. Int. J. Obes..

[25] Gangwar A., Tiwari S., Rawat A., Verma A., Singh K., Kant S., Garg R.K., Singh P.K. (2018). Circadian Preference, Sleep Quality, and Health-impairing Lifestyles Among Undergraduates of Medical University. Cureus.

[26] Muñoz J.S.G., Gallego M.G., Soler I.D., Ortega M.C.B., Cáceres C.M.M., Morante J.J.H. (2019). Effect of a chronotype-adjusted diet on weight loss effectiveness: A randomized clinical trial. Clin. Nutr..

[27] Strojny Z., Rutkowski R., Kanikowska A., Zawada A., Juchacz A., Grzymisławski M., Sato M., Litwinowicz M., Korybalska K., Bręborowicz A. (2021). No significant effect of the individual chronotype on the result of moderate calorie restriction for obesity—A pilot study. Nutrients.

[28] Broussard J.L., Cauter E. (2016). Van Disturbances of sleep and circadian rhythms: Novel risk factors for obesity. Curr. Opin. Endocrinol. Diabetes Obes..

[29] Ahmad M., Nur Syafiqa N.S.B., Tharumalay R.D., Din N.C., Ibrahim N., Amit N., Farah N.M.F., Osman R.A., Hamid M.F.A., Ibrahim I.A. (2020). The effects of circadian rhythm disruption on mental health and physiological responses among shift workers and general population. Int. J. Environ. Res. Public Health.

[30] Larsen S.C., Horgan G., Mikkelsen M.L.K., Palmeira A.L., Scott S., Duarte C., Santos I., Encantado J., Driscoll R.O., Turicchi J. (2020). Association between objectively measured sleep duration, adiposity and weight loss history. Int. J. Obes..

[31] Fenton S., Burrows T.L., Skinner J.A., Duncan M.J. (2021). The influence of sleep health on dietary intake: A systematic review and meta-analysis of intervention studies. J. Hum. Nutr. Diet..

[32] Gan W.Y., Mohd Nasir M.T., Zalilah M.S., Hazizi A.S. (2011). Differences in eating behaviours, dietary intake and body weight status between male and female Malaysian university students. Malays. J. Nutr..

[33] Manan W.A.W.M., Firdaus N.I., Safiah M.Y., Haslinda S.M.D., Poh B.K., Norimah A.K., Azmi M.Y., Tahir A., Mirnalini K., Zalilah M.S. (2012). Meal patterns of malaysian adults: Findings from the Malaysian Adults Nutrition Survey (MANS). Malays. J. Nutr..

[34] Mazri F.H., Manaf Z.A., Shahar S., Ludin A.F.M. (2020). The association between chronotype and dietary pattern among adults: A scoping review. Int. J. Environ. Res. Public Health.

[35] Lopez-Minguez J., Gómez-Abellán P., Garaulet M. (2016). Circadian rhythms, food timing and obesity. Proc. Nutr. Soc..

[36] Hirshkowitz M., Whiton K., Albert S.M., Alessi C., Bruni O., DonCarlos L., Hazen N., Herman J., Katz E.S., Kheirandish-Gozal L. (2015). National sleep foundation’s sleep time duration recommendations: Methodology and results summary. Sleep Health.

[37] Rusali R., Shahar S., Lee X.W., Abdul Manaf Z. (2016). Effectiveness of a Structured Weight Management Programme at Workplace among Employees of a Petroleum Industry in Malaysia. J. Sains Kesihat. Malays..

[38] Rusali R., Manaf Z.A., Shahar S., Mazri F.H., Ibrahim N., Ludin A.F.M., Singh D.K.A., Ali N.M. (2018). Comparison of the effectiveness of online and face-to-face weight-loss interventions in the workplace: Evidence from Malaysia. Sains Malays..

[39] Glanz K., Rimer B.K. (2005). Theory at a Glance A Guide for Helath Promotion in Practice.

[40] Department of Statistics Malaysia (DOSM) (2019). Household Income & Basic Amenities Survey Report 2019.

[41] Roenneberg T., Wirz-Justice A., Merrow M. (2003). Life between clocks: Daily temporal patterns of human chronotypes. J. Biol. Rhythm..

[42] Mazri F.H., Manaf Z.A., Shahar S., Mat Ludin A.F., Karim N.A., Ban A.Y.L., Osman R.A. (2021). Modified Munich chronotype questionnaire for application to short-interval split sleep of non-shift workers. Chronobiol. Int..

[43] Shahar S., Earland J., Rahman S.A. (2000). Validation of a dietary history questionnaire against a 7-d weighed record for estimating nutrient intake among rural elderly Malays. Malays. J. Nutr..

[44] Allison K.C., Lundgren J.D., O’Reardon J.P., Martino N.S., Sarwer D.B., Wadden T.A., Crosby R.D., Engel S.G., Stunkard A.J. (2008). The Night Eating Questionnaire (NEQ): Psychometric properties of a measure of severity of the Night Eating Syndrome. Eat. Behav..

[45] Soo K.L., Wan Abdul Manan W.M., Wan Suriati W.N. (2015). The Bahasa Melayu version of the global physical activity questionnaire: Reliability and validity study in Malaysia. Asia Pac. J. Public Health.

[46] Armstrong T., Bull F. (2006). Development of the World Health Organization Global Physical Activity Questionnaire (GPAQ). J. Public Health.

[47] Matthews D.R., Hosker J.P., Rudenski A.S., Naylor B.A., Treacher D.F., Turner R.C. (1985). Homeostasis model assessment: Insulin resistance and β-cell function from fasting plasma glucose and insulin concentrations in man. Diabetologia.

[48] Mazri F.H., Manaf Z.A., Shahar S., Mat Ludin A.F., Karim N.A., Hazwari N.D., Kek Q.W., Abdul Basir S.M., Arifin A. (2021). Do Temporal Eating Patterns Differ in Healthy versus Unhealthy Overweight/Obese Individuals?. Nutrients.

[49] Wellard-Cole L., Davies A., Allman-Farinelli M. (2021). Contribution of foods prepared away from home to intakes of energy and nutrients of public health concern in adults: A systematic review. Crit. Rev. Food Sci. Nutr..

[50] Mitchell E., Yang Q., Behr H., Ho A., DeLuca L., May C., Michaelides A. (2021). Long-Term Food Choice and Weight Loss on a Mobile Program with Food Color System. Curr. Dev. Nutr..

[51] Molina T.A., Burgess H.J. (2011). Calculating the dim light melatonin onset: The impact of threshold and sampling rate. Chronobiol. Int..

[52] Lopez-Minguez J., Saxena R., Bandín C., Scheer F.A., Garaulet M. (2018). Late dinner impairs glucose tolerance in MTNR1B risk allele carriers: A randomized, cross-over study. Clin. Nutr..

[53] McHill A.W., Phillips A.J.K., Czeisler C.A., Keating L., Yee K., Barger L.K., Garaulet M., Scheer F.A.J.L., Klerman E.B. (2017). Later circadian timing of food intake is associated with increased body fat. Am. J. Clin. Nutr..

[54] Montaruli A., Castelli L., Galasso L., Mulè A., Bruno E., Esposito F., Caumo A., Roveda E. (2019). Effect of chronotype on academic achievement in a sample of Italian University students. Chronobiol. Int..

[55] Taillard J., Sagaspe P., Philip P., Bioulac S. (2021). Sleep timing, chronotype and social jetlag: Impact on cognitive abilities and psychiatric disorders. Biochem. Pharmacol..

[56] Papandreou C., Bulló M., Díaz-López A., Martínez-González M.A., Corella D., Castañer O., Vioque J., Romaguera D., Martínez A.J., Pérez-Farinós N. (2020). High sleep variability predicts a blunted weight loss response and short sleep duration a reduced decrease in waist circumference in the PREDIMED-Plus Trial. Int. J. Obes..

[57] Garcez M.R., de Castro M.A., César C.L.G., Goldbaum M., Fisberg R.M. (2021). A chrononutrition perspective of diet quality and eating behaviors of Brazilian adolescents in associated with sleep duration. Chronobiol. Int..

[58] Tiuganji N.M., Nehme P., Marqueze E.C., Isherwood C.M., Martins A.J., Vasconcelos S., Cipolla-Neto J., Lowden A., Skene D.J., Moreno C.R.C. (2020). Eating behavior (Duration, content, and timing) among workers living under different levels of urbanization. Nutrients.

[59] Chan C.M.H., Siau C.S., Wong J.E., Wee L.H., Jamil N.A., Hoe V.C.W. (2021). Prevalence of insufficient sleep and its associated factors among working adults in Malaysia. Nat. Sci. Sleep.

[60] Ross K.M., Graham Thomas J., Wing R.R. (2016). Successful weight loss maintenance associated with morning chronotype and better sleep quality. J. Behav. Med..

[61] Kühnle T., Grupe G., Foitzik S., Cremer T., Haszprunar G., Roennenberg T. (2006). Quantitative Analysis of Human Chronotypes. Ph.D. Thesis.

