# Supplementary material for: Development and Evaluation of Integrated Chrono-Nutrition Weight Reduction Program among Overweight/Obese with Morning and Evening Chronotypes
Source: Int J Environ Res Public Health. 2022 Apr 7;19(8):4469. doi: 10.3390/ijerph19084469 (PMC9029858; doi:10.3390/ijerph19084469)
Supplement: Supplementary file 1 [file ijerph-19-04469-s001.zip › ijerph-1607748-supplementary.pdf]

Table S1: Pre-post intervention differences in total dietary intake, meal timing, NES score, sleep habits and physical activity (Complete case, n=85)

	MT (n=44)		ET (n=41)		Mean difference (95% CI)		
	Pre	Post	Pre	Post	Time	CT	Time x CT
Total dietary intake							
EI (kcal/day)	1684 ± 443	1227 ± 331	1889 ± 477	1334 ± 394	-506 (-594, -417)***	-156 (-311, -0.8)*	-98 (-275, 80)
CHO (g/day)	207.0 ± 64.3	152.7 ± 46.0	224.9 ± 66.2	160.5 ± 48.6	-59.3 (-72.0, -46.6)***	-12.8 (-33.9, 8.2)	9.8 (-15.6, 35.3)
% E CHO	49.1 ± 5.7	49.7 ± 6.2	47.6 ± 5.7	48.6 ± 6.1	0.8 (-0.8, 2.3)	1.4 (-0.7, 3.4)	-0.5 (-3.5, 2.6)
Protein (g/day)	64.6 ± 16.6	57.6 ± 10.5	71.8 ±17.6	60.1 ± 17.4	-9.3 (-12.8, -5.7)***	-4.8 (-10.6, 1.0)	4.7 (-11.8, 2.4)
% E protein	15.6 ± 3.0	19.5 ± 3.8	15.3 ± 1.7	18.4 ± 4.1	3.5 (2.5, 4.5)***	0.7 (-0.4, 1.7)	0.8 (-1.2, 2.8)
Fat (g/day)	66.0 ± 19.2	42.9 ± 16.9	78.0 ± 22.9	50.2 ± 20.5	-25.4 (-30.1, -20.7)***	-9.7 (-16.9, -2.4)**	4.8 (-4.6, 14.2)
% E fat	35.1 ± 5.0	30.8 ± 6.3	37.1 ± 5.1	33.0 ± 6.6	-4.2 (-5.7, -2.6)***	-2.1 (-4.1, -0.2)*	-0.2 (-3.2, 2.9)
Meal timing							
First meal (hh:mm)	08:12 ± 0:41	08:03 ± 0:47	08:27 ± 0:41	08:25 ± 0:40	-0.1 (-0.2, 0.0)	-0.3 (-0.6, -0.0)*	-0.1 (-0.4, 0.1)
Last meal (hh:mm)	19:56 ± 1:15	19:36 ± 1:08	20:23 ± 1:41	20:07 ± 1:30	-03 (-0.6, 2.3)	-0.5 (-1.0, 0.0)	-0.1 (-0.8, 0.6)
Total eating window (h)	11.7 ± 1.6	11.6 ± 1.3	11.9 ± 1.7	11.7 ± 1.5	-0.2 (-0.6, 0.2)	-0.2 (-0.7, 0.4)	0.0 (-0.7, 0.8)
Midpoint eating (hh:mm)	14:04 ± 0:38	13:50 ± 0:42	14:25 ± 0:59	14:16 ± 0:53	-0.2 (-0.4, -0.0)*	-0.4 (-0.7, -0.1)*	-0.1 (-0.4, 0.3)
Elapse SOn-last meal (h)	3.0 ± 1.5	3.5 ± 1.3	3.5 ± 1.8	3.2 ± 1.5	0.1 (-0.3, 0.5)	-0.1 (-0.7, 0.4)	0.8 (0.1, 1.6)*
NES score	10.5 ± 4.9	9.3 ± 4.1	10.9 ± 5.4	8.9 ± 5.0	-1.6 (-2.7, -0.6)**	-0.0 (-1.8, 1.8)	0.8 (-1.3, 3.0)
Sleep							
Work SD (hour)	6.6 ± 1.0	6.7 ± 0.9	6.1 ± 0.9	7.4 ± 1.2	0.7 (0.4, 1.0)***	-0.1 (-0.4, 0.2)	-1.1 (-1.7, -0.6)***
Free SD (hour)	7.3 ± 1.2	6.5 ± 0.9	6.7 ± 1.5	6.7 ± 0.8	-0.4 (-0.7, -0.1)*	0.2 (-0.2, 0.6)	-0.8 (-1.4, -0.2)*
Average SOn (hh:mm)	22:57 ± 0:48	23:06 ± 0:48	23:55 ± 0:53	23:18 ± 0:55	-0.2 (-0.4, -0.1)*	-0.6 (-0.9, -0.3)**	0.8 (0.4, 1.1)***
Average SOff (hh:mm)	05:48 ± 0:31	05:40 ± 0:29	06:13 ± 0:38	06:15 ± 0:46	-0.1 (-0.2, 0.1)	-0.5 (-0.7, -0.3)***	-0.2 (-0.4, 0.1)
Average SD (hour)	6.8 ± 0.9	6.6 ± 0.8	6.3 ± 1.0	6.9 ± 0.8	0.2 (0.0, 0.4)*	0.1 (-0.2, 0.4)	-0.8 (-1.2, -0.4)***
Social jetlag (min)	17.6 ± 30.2	23.4 ± 23.5	54.5 ± 44.4	39.6 ± 42.0	-4.6 (-13.9, 4.8)	-26.5 (-38.9, -14.0)***	20.6 (2.1, 39.1)
Physical activity (MET)	1663.7 ± 1710.5	3467.9 ± 2386.9	1718.6 ± 2172.7	3558.1 ± 2520.1	1821.8 (1240.4, 2403.3)***	-72.5 (-839.0, 694.0)	-35.2 (-1198.1, 1127.7)

Data were presented in mean and standard deviation. Indicated statistically significant using Two-way Mixed ANOVA test at *p<0.05, **p<0.01 and ***p<0.001.
Abbreviations: MT, morning chronotype; ET, evening chronotype; CI, confidence interval; CT, chronotype; EI, energy intake; CHO, carbohydrate; % E, percentage energy; hh:mm, local time in hour and minute; h, hour; min, minutes; Elapse SOn-last meal, elapse time between last mealtime and sleep onset; NES, night eating syndrome; SD, sleep duration

Table S2 (a): Distribution of % total energy and energy from macronutrients in early and late windows (n=91)

	M-type (n=46)		E-type (n=45)		P value		
	**Pre**	**Post**	**Pre**	**Post**	**Time**	**Group**	**Time x Group**
			**Early window**				
% E total	64.8 ± 9.6	67.2 ± 9.5	62.7 ± 8.9	65.6 ± 12.7	**0.028**	0.308	0.828
% E CHO	31.9 ± 6.5	33.5 ± 5.7	29.5 ± 6.4	33.0 ± 8.8	**0.002**	0.217	0.286
% E protein	10.3 ± 3.2	13.3 ± 3.9	9.9 ± 1.9	12.0 ± 3.8	**<0.001**	0.084	0.307
% E fat	22.6 ± 3.9	20.4 ± 5.4	23.3 ± 5.4	20.6 ± 5.1	**<0.001**	0.589	0.698
**Breakfast**							
% E total	28.3 ± 7.5	24.5 ± 7.9	27.0 ± 10.5	25.2 ± 11.4	**0.004**	0.841	0.332
% E CHO	14.2 ±4.7	12.6 ± 4.8	12.6 ± 5.9	13.1 ± 6.8	0.307	0.616	**0.048**
% E protein	4.1 ± 1.9	4.3 ± 2.0	4.1 ± 2.0	4.3 ± 2.5	0.458	1.000	0.588
% E fat	10.0 ± 4.0	7.6 ± 3.8	10.3 ± 4.7	7.8 ± 3.6	**<0.001**	0.739	0.969
**Morning snack**							
%E total	3.0 ± 5.0	3.7 ± 4.9	2.9 ± 4.6	2.9 ±6.9	0.593	0.635	0.641
% E CHO	2.0 ± 3.0	2.3 ± 2.9	1.7 ± 2.5	1.8 ± 4.0	0.534	0.447	0.740
% E protein	0.3 ± 1.0	0.4 ± 0.6	0.2 ± 0.5	0.2 ± 0.8	0.954	0.419	0.600
% E fat	0.7 ± 1.5	1.0 ± 1.8	1.0 ± 1.7	0.9 ± 2.3	0.633	0.929	0.384
**Lunch**							
% E total	33.5 ± 10.3	39.0 ± 10.2	32.8 ± 8.7	37.5 ± 11.2	**<0.001**	0.546	0.770
% E CHO	15.7 ± 5.6	18.7 ± 6.2	15.3 ± 4.9	18.1 ± 6.7	**<0.001**	0.598	0.925
% E protein	5.9 ± 2.6	8.6 ± 3.2	5.5 ± 1.6	7.5 ± 2.5	**<0.001**	0.062	0.227
% E fat	11.9 ± 4.0	11.7 ± 4.3	12.0 ± 4.0	11.9 ± 5.0	0.772	0.811	0.970
			**Late window**				
% E total	35.2 ± 9.6	32.8 ± 9.5	37.3 ± 8.9	34.4 ± 12.7	**0.028**	0.308	0.828
% E CHO	17.3 ± 5.4	16.0 ± 5.9	18.2 ± 5.7	15.7 ± 5.9	**0.005**	0.599	0.269
% E protein	5.6 ± 2.0	6.2 ± 2.5	5.6 ± 1.7	6.2 ± 2.8	**0.006**	0.904	0.972
% E fat	12.3 ± 4.9	10.6 ± 4.4	13.5 ± 4.2	12.5 ± 6.7	**0.042**	0.076	0.580
**Afternoon snack**							
% E total	5.6 ± 6.6	4.2 ± 5.8	5.3 ± 6.8	2.7 ± 4.4	**0.035**	0.444	0.576
% E CHO	3.2 ± 4.0	2.4 ± 2.6	3.4 ± 4.6	1.8 ± 2.7	**0.021**	0.823	0.560
% E protein	0.5 ± 0.6	0.4 ± 1.2	0.5 ± 0.6	0.1 ± 0.4	0.256	0.394	0.346
% E fat	1.9 ± 2.2	1.4 ± 2.7	1.4 ± 1.8	0.8 ± 1.8	0.092	0.155	0.836
**Dinner**							
% E total	29.6 ± 9.1	28.6 ± 11.0	32.0 ± 9.6	31.7 ± 13.3	0.594	0.159	0.771
% E CHO	14.1 ± 5.1	13.6 ± 6.6	14.8 ±5.0	13.9 ± 6.1	0.279	0.608	0.698
% E protein	5.1 ± 2.0	5.8 ± 2.6	5.1 ± 1.9	6.1 ± 2.8	**0.002**	0.735	0.610
% E fat	10.4 ± 4.5	9.2 ± 4.1	12.1 ±4.4	11.7 ± 6.7	0.248	**0.015**	0.550

Bold P value indicate significant difference between pre- and post-intervention within each chronotypes by using Two-way Mixed ANOVA test.

Supplement Table 2(b): Distribution of % total energy and energy from macronutrients in early and late windows (Complete case, n=85)

	M-type (n=44)		E-type (n=41)		P value		
	**Pre**	**Post**	**Pre**	**Post**	**Time**	**Group**	**Time x Group**
			**Early window**				
% E total	64.6 ± 9.7	67.1 ± 9.7	62.8± 9.1	66.0 ± 9.4	**0.027**	0.438	0.786
% E CHO	31.9 ± 6.6	33.7 ± 5.8	29.3 ± 6.6	33.1 ± 9.2	**0.002**	0.227	0.251
% E protein	10.3 ± 3.2	13.3 ± 3.9	9.9 ± 1.9	12.0 ± 3.8	**<0.001**	0.152	0.371
% E fat	22.5 ± 3.9	20.2 ± 5.2	23.6 ± 5.2	20.6 ± 5.5	**<0.001**	0.386	0.627
**Breakfast**							
% E total	28.1 ± 7.4	24.1 ± 7.6	27.8 ± 10.5	25.7 ± 11.7	**0.004**	0.822	0.367
% E CHO	14.1 ±4.7	12.5 ± 4.6	12.8 ± 6.0	13.4 ± 6.9	0.364	0.890	0.051
% E protein	4.0 ± 1.9	4.3 ± 1.7	4.3 ± 2.0	4.3 ± 2.5	0.466	0.714	0.601
% E fat	9.9 ± 4.0	7.4 ± 3.8	10.7 ± 4.7	8.0 ± 3.7	**<0.001**	0.296	0.871
**Morning snack**							
%E total	3.1 ± 5.1	3.8 ± 5.0	2.9 ± 4.8	3.0 ± 7.2	0.601	0.611	0.651
% E CHO	2.0 ± 3.0	2.4 ± 2.9	1.7 ± 2.6	1.9 ± 4.2	0.539	0.474	0.751
% E protein	0.4 ± 1.0	0.4 ± 0.6	0.2 ± 0.5	0.3 ± 0.8	0.944	0.335	0.600
% E fat	0.8 ± 1.5	1.1 ± 1.7	1.0 ± 1.8	0.9 ± 2.4	0.648	0.966	0.391
**Lunch**							
% E total	33.4 ± 10.1	39.2 ± 10.0	32.0 ± 8.4	37.3 ± 11.4	**<0.001**	0.348	0.848
% E CHO	15.7 ± 5.7	18.8 ± 6.2	14.8 ± 4.7	17.9 ± 6.8	**<0.001**	0.340	0.998
% E protein	5.8 ± 2.5	8.6 ± 3.2	5.4 ± 1.6	7.5 ± 2.6	**<0.001**	0.080	0.294
% E fat	11.9 ± 3.9	11.7 ± 4.2	11.9 ± 4.1	11.7 ± 5.2	0.772	0.986	0.965
			**Late window**				
% E total	35.4 ± 9.7	32.9 ± 9.7	37.2 ± 9.0	34.0 ± 13.0	**0.027**	0.438	0.786
% E CHO	17.3 ± 5.4	16.0 ± 5.8	18.5 ± 5.8	15.4 ± 5.9	**0.004**	0.770	0.239
% E protein	5.4 ± 1.9	6.2 ± 2.5	5.5 ± 1.7	6.3 ± 2.9	**0.006**	0.752	0.918
% E fat	12.5 ± 4.9	10.8 ± 4.4	13.6 ± 4.3	12.5 ± 7.0	**0.043**	0.134	0.615
**Afternoon snack**							
% E total	5.7 ± 6.7	4.3 ± 5.8	5.6 ± 7.0	3.0 ± 4.6	**0.033**	0.492	0.540
% E CHO	3.3 ± 4.0	2.4 ± 2.6	3.6 ± 4.8	2.0 ± 2.8	**0.020**	0.868	0.521
% E protein	0.5 ± 0.6	0.5 ± 1.2	0.5 ± 0.6	0.3 ± 0.4	0.247	0.392	0.332
% E fat	1.9 ± 2.2	1.4 ± 2.7	1.6 ± 1.9	0.9 ± 1.8	0.091	0.195	0.804
**Dinner**							
% E total	29.6 ± 9.2	28.5 ± 11.1	31.6 ± 9.7	31.2 ± 13.7	0.599	0.247	0.781
% E CHO	14.1 ± 4.9	13.6 ± 6.6	14.6 ± 4.8	13.5 ± 6.0	0.275	0.801	0.678
% E protein	4.9 ± 1.9	5.7 ± 2.6	5.0 ± 1.9	6.1 ± 2.8	**0.002**	0.590	0.557
% E fat	10.5 ± 4.6	9.3 ± 4.4	12.0 ±4.4	11.6 ± 6.9	0.254	**0.037**	0.569

Bold P value indicate significant difference between pre- and post-intervention within each chronotypes by using Two-way Mixed ANOVA test.

Table S3: Pre-post intervention differences in adiposity, and biochemical parameters (Complete case, n=85).

	**MT (n=44)**		**ET (n=41)**		**Mean difference (95% CI)**	
	**Pre**	**Post**	**Pre**	**Post**	**Time**	**Time x CT**
Adiposity						
Weight (kg)	79.7 ± 16.5	75.3 ± 15.6	80.6 ± 13.5	76.7 ± 12.3	-4.2 (-5.2, -3.2)***	-0.5 (-2.4, 1.5)
BMI (kg/m^2)	31.3 ± 4.4	29.6 ± 4.6	30.8 ± 4.0	29.3 ± 3.9	-1.6 (-2.0, -1.3)***	-0.2 (-0.9, 0.5)
Body fat (%)	41.4 ± 7.5	39.1 ± 8.6	39.3 ± 7.8	37.4 ± 8.2	-2.1 (-2.7, -1.5)***	-0.3 (-1.5, 0.9)
WC (cm)	94.0 ± 11.8	90.2 ± 11.8	93.4 ± 9.5	88.9 ± 8.6	-4.2 (-5.2, -3.1)***	0.7 (-1.4, 2.8)
Biochemical						
FBG (mmol/L)	5.0 ± 0.5	4.9 ± 0.6	5.0 ± 0.7	5.0 ± 0.8	-0.1 (-0.2, 0.03)	-0.01 (-0.2, 0.2)
Insulin (µIU/ml)	14.0 ± 7.5	10.2 ± 6.9	13.0 ± 9.6	9.9 ± 5.9	-3.4 (-4.9, -2.0)***	-0.7 (-3.5, 2.2)
HbA1c (%)	5.8 ± 0.5	5.8 ± 0.7	5.8 ± 0.6	5.8 ± 0.5	0.01 (-0.1, 0.1)	0.1 (-0.1, 0.2)
HOMA-IR	3.2 ± 1.8	2.3 ± 2.0	3.0 ± 2.5	2.3 ± 1.6	-0.8 (-1.2, -0.4)***	-0.1 (-0.8, 0.6)
TC (mmol/L)	5.2 ± 1.0	5.2 ± 1.1	5.2 ± 0.9	5.1 ± 0.9	-0.1 (-0.2, 0.1)	0.1 (-0.2, 0.4)
TG (mmol/L)	1.3 ± 0.8	1.2 ± 0.8	1.4 ± 0.8	1.3 ± 0.9	-0.1 (-0.2, -0.01)*	-0.01 (-0.2, 0.2)
LDL-C (mmol/L)	3.3 ± 0.94	3.4 ± 1.0	3.2 ± 0.9	3.2 ± 0.8	0.04 (-0.1, 0.2)	0.1 (-0.2, 0.3)
HDL-C (mmol/L)	1.3 ± 0.3	1.3 ± 0.3	1.4 ± 0.3	1.3 ± 0.2	-0.05 (-0.1, -0.01)*	0.03 (-0.1, 0.1)
Uric acid (mmol/L)	0.4 ± 0.1	0.3 ± 0.1	0.4 ± 0.1	0.4 ± 0.1	-0.01 (-0.03, -0.00)*	0.00 (-0.02, 0.02)
Blood pressure						
Systolic (mmHg)	129.0 ± 10.3	119.0 ± 12.1	135.5 ± 12.3	127.4 ± 14.5	-9.1 (-11.4, -6.7)***	-1.9 (-6.6, 2.8)
Diastolic (mmHg)	80.6 ± 9.2	75.4 ± 12.3	81.4 ± 9.3	77.4 ± 9.9	-4.6 (-7.1, -2.2)***	-1.3 (-6.2, 3.6)

Data were presented in mean and standard deviation. Indicated statistically significant using Two-way Mixed ANOVA test at *p<0.05, **p<0.01 and ***p<0.001. Abbreviations: MT, morning chronotype; ET, evening chronotype; CI, confidence interval; CT, chronotype; BMI, body mass index; WC, waist circumference; FBG, fasting blood glucose; HOMA-IR, homeo-static model assessment of insulin resistance; TC, total cholesterol; LDL-C, low-density lipoprotein cholesterol; HDL-C, high-density lipoprotein cholesterol.

Table S4: Eating out (food-away from home) frequency in a week (n=91)

	**Pre**	**Post**	**P-value**
Breakfast	4.2 ± 2.7	2.3 ± 2.5	**<0.001**
Morning snack	1.1 ± 2.0	0.4 ± 1.3	**0.005**
Lunch	4.0 ± 2.4	3.8 ± 2.4	0.496
Afternoon snack	1.6 ± 2.2	0.5 ± 1.1	**<0.001**
Dinner	2.3 ± 2.7	1.6 ±2.0	**0.020**

Bold P value indicate significant difference between pre- and post-intervention by using paired T-test.
